# The representational nature of action–effect relations: A memory process dissociation approach

**DOI:** 10.3758/s13423-025-02794-3

**Published:** 2026-01-14

**Authors:** Marcel R. Schreiner, Wilfried Kunde

**Affiliations:** Department of Psychology III, Julius-Maximilians-Universität Würzburg, Röntgenring 11, Würzburg, 97070 Germany

**Keywords:** Action–effect learning, Action control, Explicit memory, Implicit memory, Process dissociation, Cognitive modeling

## Abstract

Learning how actions change the environment is crucial for goal-directed actions and skill acquisition. Here, we applied a process dissociation approach to investigate the contribution of explicit and implicit memory to the learning of action–effect relations across four experiments. Participants produced object images by pressing one of two keys, with each action–effect episode experienced three times. Learning was either incidental (Experiments 1-2) or intentional (Experiments 2-4) and occurred under full (Experiments 1-4) or divided (Experiments 3-4) attention. In a test phase, participants were re-presented the effect images and asked to either reproduce or alternate the action that had produced them. Results obtained through cognitive modeling revealed that action–effect relations are primarily represented in explicit memory, with minimal contributions of implicit memory. Intentional learning enhanced memory compared to incidental learning, while divided attention during encoding reduced it, with these factors mainly affecting explicit memory. These findings elucidate the mechanisms underlying skill acquisition and provide insights into the representational nature of action–effect relations.

## Introduction

Through our actions, we interact with our environment, causing perceivable effects. Yet, to produce such effects on purpose, and thus to act in a goal-oriented manner, it is crucial that we learn how actions and subsequent environmental effects are related (Altmann & Trafton, 2002; Liesner et al., 2020). Thus, some form of storage of linkages between proximal body-related (motor activity) and distal environment-related (perceptual changes) features is required. The acquisition of such action–effect linkages has been repeatedly demonstrated (Elsner & Hommel, 2001; Frings et al., 2020, 2024a; Hommel et al., 2001; Ziessler et al., 2004).

While linkages between action and effect features have largely been assumed to be short-lived (Dutzi & Hommel, 2009; Frings, 2011; Frings et al., 2022; Moeller & Frings, 2022; Pastötter et al., 2021), they would need to be more sustained to serve adaptive functions. There is indeed evidence for enduring storage of action–effect relations, suggesting their representation in long-term memory (Elsner & Hommel, 2001; Frings et al., 2024b; Hommel et al., 2003; Schreiner & Kunde, 2025). However, the exact nature of these representations is largely unclear, pertaining to a more general problem across various domains of psychological research (Kaup et al., 2024).

Long-term memory can be subdivided into explicit and implicit memory (Graf & Schacter, 1985; Squire, 2004, 2009; Tulving, 1972). Whereas representations in explicit memory are consciously accessible, can be intentionally used, and require attentional resources, representations in implicit memory are not consciously accessible, exert automatic influences on behavior, and are largely attention invariant (Graf & Schacter, 1985; Jacoby, 1991; Schacter, 1992; Squire, 2004; Yonelinas, 2002). It has been the dominant assumption that action–effect relations are represented in implicit memory, especially in research inspired by ideomotor theory (Greenwald, 1970; Hommel et al., 2001; James, 1890). Indeed, several pieces of evidence support this assumption (Elsner & Hommel, 2001; Kunde, 2004; Kunde & Janczyk, 2024; Le Bars et al., 2016). However, recent evidence has emerged that suggests that action–effect relations are represented in explicit memory (Custers, 2023; Janczyk et al., 2024; Schreiner et al., 2025a; Sun et al., 2020). Thus, there is considerable ambiguity regarding the nature of representations supporting the learning of action–effect relations. Nevertheless, memory processes contributing to the learning of action–effect relations have, to our knowledge, never been directly dissociated.

The present research aims to address the ambiguity around the representational nature of action–effect relations in a novel and more direct way. To disentangle the contribution of explicit and implicit memory processes, we adapted the process dissociation procedure (Jacoby, 1991, 1998). This procedure is based on the logic of opposition, which posits that explicit and implicit memory processes can be disentangled by creating conditions where responses driven by automatic response tendencies diverge from those resulting from conscious reasoning (Jacoby, 1991). In an initial learning phase, participants could produce effects (images of everyday objects) by pressing a left or right key in a forced-choice task. Each action–effect episode was experienced three times. In a later test phase, participants were re-presented with the effects. Crucially, in an *inclusion condition*, participants were instructed to reproduce the corresponding action. In this case, automatic response tendencies elicited by implicit memory traces and conscious retrieval of the associated action elicited by explicit memory traces result in the same response at test. In a separate *exclusion condition*, participants were instructed to retrieve the corresponding action and then respond with the *other* action (e.g., press the right key if they previously produced the effect by pressing the left key). In this case, automatic response tendencies elicited by implicit memory traces would push participants towards repeating the action, whereas conscious retrieval of the associated action elicited by explicit memory traces would push participants towards alternating the action. To statistically distinguish between the contribution of explicit and implicit memory processes, we employed cognitive modeling, specifically, multinomial processing tree (MPT) models (Batchelder & Riefer, 1999; Erdfelder et al., 2009; Riefer & Batchelder, 1988). Model parameters reflect the assumed cognitive processes that cause participants to respond in a certain way.

The contribution of explicit and implicit memory to the (early) learning of action–effect relations was investigated in four experiments. In Experiment 1, participants performed the task under incidental learning. Given the ambiguity of empirical evidence regarding the representational nature of action–effect relations, Experiment 1 was largely exploratory, although we tentatively expected a stronger contribution of explicit than implicit memory. In Experiment 2, we distinguished between incidental and intentional learning. Considering that explicit memory enables one to make intentional use of a memory and rests on attentional resources, we expected intentional learning to increase the contribution of explicit memory. In Experiments 3 and 4, participants intentionally learned action–effect relations under full or divided attention. Considering that explicit memory requires attentional resources, whereas implicit memory is largely attention invariant, we expected divided attention to reduce the contribution of explicit memory, and do so with a greater extent than is the case for implicit memory.

## Experiment 1: Incidental Learning

In Experiment 1, participants incidentally learned action–effect relations, that is, they were not instructed to remember the effect images and corresponding actions, and were not informed about the later memory test.

### Methods

The task was conducted under incidental learning conditions, with two test conditions (inclusion and exclusion). The experiment was implemented using lab.js (Henninger et al., 2022) and conducted online. Data collection was managed by JATOS (Lange et al., 2015). Analyses were conducted in R 4.4.0 (R Core Team, 2024). We used the R packages *papaja 0.1.2* (Aust & Barth, 2023) and *tinylabels 0.2.4* (Barth, 2023) for reporting.

**Fig. 1:**
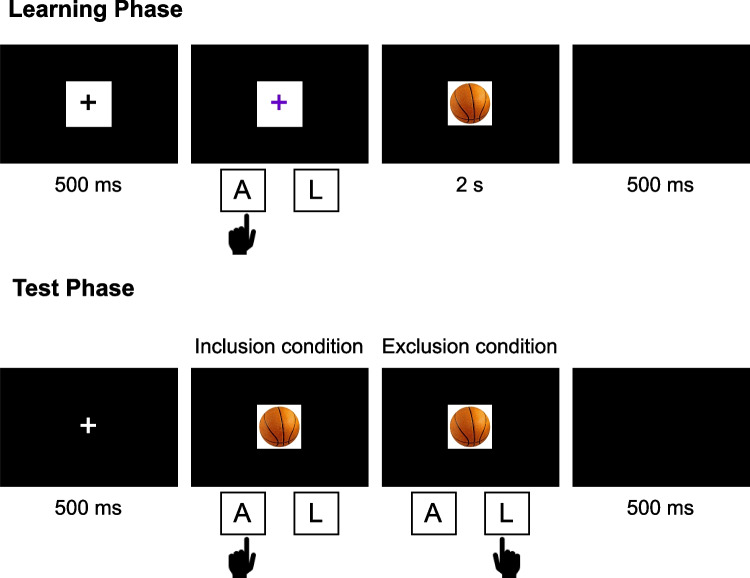
Basic experimental procedure with an example learning and test trial. *Notes.* In the learning phase, participants were first presented with a fixation cross within a white box. The cross then changed color to either purple, demanding an A (left) key press, or blue, demanding an L (right) key press (the action). After pressing the correct key, an object image appeared inside the box (the effect). If an incorrect key was pressed, participants received error feedback, and the trial was repeated. Each trial occurred three times (not counting trials with an incorrect key press). In the test phase, participants were first presented with a fixation cross, followed by an object image. In the inclusion condition, they had to press the same key through which they produced the image in the learning phase (i.e., repeat the corresponding action). In the exclusion condition, they had to press the key through which they did not produce the image in the learning phase (i.e., alternate the corresponding action)

#### Participants

Participants were recruited via the Prolific panel (https://www.prolific.com) and received a compensation of £2.10 for the approximately 14-min experiment. They were prescreened to speak English fluently and to have normal or corrected-to-normal vision and no color vision deficiency. A simulation-based a priori power analysis conducted in multiTree v0.47 (Moshagen, 2010) for detecting a difference of .08 between the explicit ($E$) and implicit ($I$) memory parameters with 80% power given a significance level of 5% yielded a desired sample size of 34 participants per test condition. To account for possible data exclusions, we collected data from 40 participants per condition (*N* = 80). Two participants were excluded due to experiencing technical problems, resulting in a final sample size of *N* = 78 participants (43 participants in the inclusion condition and 35 in the exclusion condition). Participants were on average 27.88 years old (*SD* = 6.51, range = 18–47), 38 participants were women, 39 were men, and one was non-binary, 75 participants were right-handed, one was left-handed, and two were ambidextrous.

#### Design

The experiment employed a one-factorial (test condition: inclusion vs. exclusion) between-participants design. In the inclusion condition, participants were instructed to reproduce at test the action through which they produced a presented effect image in the learning phase (e.g., press the A key if they produced the effect by pressing the A key in the learning phase). In the exclusion condition, participants were instructed to retrieve the corresponding action but then respond with the *other* action (e.g., press the L key if they produced the effect by pressing the A key in the learning phase). Participants were randomly assigned to the experimental conditions.

#### Materials

Stimuli were 40 images of everyday objects (e.g., a basketball, see Fig. 1) taken from the bank of standardized stimuli (BOSS, Brodeur et al., 2010, 2014). They were selected to have mean visual complexity ratings smaller than 2, mean familiarity and object agreement ratings greater than 4, and mean viewpoint agreement ratings greater than 2.5 (rated on a Likert scale ranging from 1 to 5). An additional four object images were used as practice stimuli. These stimuli were used as effects that participants could produce through a key press action.

**Fig. 2:**
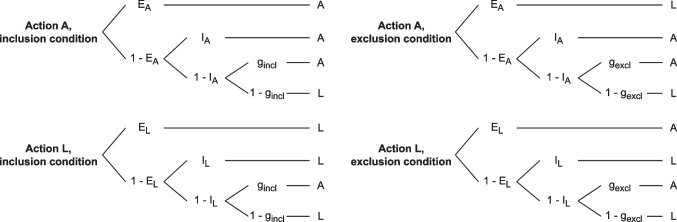
Diagram of the basic multinomial processing tree model as used in Experiment 1 and extended in Experiments 2-4. *Notes.* Model parameters: $E_A$ = probability to retrieve action A from explicit memory, $E_L$ = probability to retrieve action L from explicit memory, $I_A$ = probability to retrieve action A from implicit memory, $I_L$ = probability to retrieve action L from implicit memory, $g_{incl}$ = probability to guess and respond with action A in the inclusion condition, $g_{excl}$ = probability to guess and respond with action A in the exclusion condition. Response categories: A = respond with action A (left key press), L = respond with action L (right key press)

#### Procedure

The experiment consisted of a learning and a test phase, similar to the induction paradigm (Elsner & Hommel, 2001). The procedure is shown in Fig. 1. In the learning phase, participants performed a forced-choice effect-production task. In each trial, participants were first presented with a white box on a black background presented in the screen center, with a black fixation cross in its center. After 500 ms, the fixation cross changed color. The color instructed participants to perform a specific action (press a left or right key). A purple cross indicated that they should press the A key with their left index finger. A blue cross indicated that they should press the L key with their right index finger. The required action was counterbalanced across trials (i.e., each action occurred 20 times across the 40 action–effect episodes). If participants performed the wrong action, they received error feedback for 1 s and the trial was repeated. If they performed the correct action, the box was substituted by an object image (the effect), which was displayed for 2 s. Participants were told beforehand that they could generate these images with their correct action. Each trial ended with a 500-ms intertrial interval during which a blank screen was shown. Each trial (i.e., action–effect episode) was repeated three times, with the constraint that identical trials could not appear subsequently (except for trials repeated due to an incorrect action). Besides this constraint, the trial order was randomized. The learning phase therefore encompassed 120 trials in total (with 40 effect images, each being produced three times through the same action). The required action was counterbalanced across trials, that is, in half of the trials participants were required to press the A key and in the other half of trials they were required to press the L key. The learning phase was preceded by four practice trials (not repeated) to familiarize participants with the procedure and train the correct color-action mapping. Participants received no instructions to remember the effect images and corresponding actions and were not informed that they would later be tested (incidental learning).

After completion of the learning phase, the test phase began. In the test phase, participants were re-presented with the effect images and were asked to remember the action they used to produce them in the learning phase. They were either instructed to reproduce the action (inclusion condition) or alternate the action (i.e., respond with the other action than the one used to produce the effect image, exclusion condition). Therefore, a correct response was an action repetition in the inclusion condition and an action alternation in the exclusion condition. To ensure that participants understood the instructions, they needed to answer two questions asking which key they should press if they were presented an image they previously generated by pressing the A or L key. They had to reread the instructions and revisit the questions until they answered both questions correctly. Participants were further instructed to be as accurate as possible on the task. Each trial began with a white fixation cross on a black background presented in the screen center for 500 ms. Then, an object image was presented, and participants had to respond according to their test condition. After pressing the A or L key, the image disappeared and the trial ended after an intertrial interval of 500 ms during which a blank screen was shown. Each of the effect images from the learning phase was presented in the test phase, resulting in 40 test trials. The trial order was randomized. Before the test phase, participants conducted two practice trials (one per action) to familiarize themselves with the procedure. After the test phase, participants received feedback regarding their performance (the percentage of correct responses).

At the end of the experiment, we collected some demographic information (age, gender, and handedness). Participants could further indicate whether there were reasons their data should be excluded from the analyses and give general comments regarding the study. Finally, they were thanked and debriefed.

#### Data analysis

Given that there were two possible responses (A or L) in the test phase, two possible actions (A or L) in the learning phase, and two experimental conditions (inclusion and exclusion), this results in eight response categories. We aggregated response frequencies across trials and participants, and analyzed these aggregated response frequencies using a multinomial processing tree (MPT) model (Batchelder & Riefer, 1999; Erdfelder et al., 2009; Riefer & Batchelder, 1988). The model is displayed in Fig. 2 and consisted of four trees, one per action in the learning phase, crossed with experimental condition.^1^ It comprises six parameters, which reflect assumed cognitive processes leading to responses falling into a specific response category. If an effect was produced by action A (a left key press), participants can retrieve an explicit memory trace with probability $E_A$, resulting in repeating action A in the inclusion condition and alternating the action (responding with L) in the exclusion condition. If explicit retrieval of a memory trace is unsuccessful with probability $1-E_A$, participants can still retrieve an implicit memory trace with probability $I_A$, resulting in repeating action A in both the inclusion and exclusion conditions. If retrieval of an implicit memory trace is also unsuccessful with probability $1-I_A$, participants can guess a response and respond with A with probability $g_{incl}$ (inclusion condition) or $g_{excl}$ (exclusion condition), and with L with probability $1-g_{incl}$ (inclusion condition) or $1-g_{excl}$ (exclusion condition). These guessing parameters therefore allow to account for a biased selection of response keys.

To make the model identifiable, we imposed theoretically derived restrictions on the model parameters. First, we applied equality constraints on parameters $E_A$ and $E_I$, and on parameters $I_A$ and $I_L$, thus assuming action invariance (i.e., no difference in explicit and implicit memory between actions A and L) and estimating only one parameter for explicit ($E$) and implicit ($I$) memory retrieval. Second, we fixed $g_{excl}$ to the stochastic guessing probability of .5 given two possible responses in the test phase, thus assuming unbiased guessing in the exclusion condition.^2^ Note that the model includes a built-in equality constraint on parameters $E$ and $I$ between the inclusion and exclusion test conditions (cf. Jacoby, 1998), which is necessary for the estimation of these parameters and the application of the process dissociation procedure.

To test whether the contribution of explicit and implicit memory to the learning of action–effect relations differed, we fit a separate model with an equality constraint on parameters $E$ and $I$, thus assuming an equal contribution of explicit and implicit memory. As the model is nested within the baseline model without this restriction, we can test whether this restrictions leads to a significantly worse model fit by evaluating the difference in the goodness-of-fit statistics ($\Delta G^2$) of the two models, which under the null hypothesis follows a $\chi ^2$ distribution with 1 degree of freedom, via a conditional likelihood-ratio test. We further tested whether there was general evidence for the contribution of explicit and implicit memory processes in the same way, by fitting nested models in which either $E$ or $I$ were restricted to zero, and testing whether these restrictions led to significantly worse model fit. The MPT model analyses were conducted using the R package *MPTinR 1.14.1* (Singmann & Kellen, 2013). We used a significance level of 5% for all tests.

Note that the aggregated MPT analysis approach we employed assumes that there is no person heterogeneity in responses (Matzke et al., 2015). Bayesian hierarchical MPT model analysis allows for analyzing data based on individual response frequencies and to account for person heterogeneity (Arnold et al., 2019; Klauer, 2006). However, such an analysis approach does not allow the joint estimation of parameters across different between-participant conditions. This is, however, crucial for the process dissociation procedure, as the dissociation of explicit and implicit memory contributions requires the joint consideration of the inclusion and exclusion test conditions, and the estimation procedure rests on the assumption that the $E$ and $I$ parameters are equal across these conditions (cf. Jacoby, 1998). In addition, a hierarchical Bayesian MPT model needs to be identified within each between-participants conditions, which would have required additional parameter constraints, resulting in a saturated model, preventing us from evaluating the model fit of the baseline model. To circumvent these problems with applying Bayesian hierarchical MPT models in the context of the current research, the inclusion and exclusion test conditions would have needed to be manipulated within participants. However, this would have entailed a switch in response mappings at test. We considered the potential cost of such changes in response mappings to outweigh the potential benefits of being able to model person heterogeneity, and thus opted for an MPT analysis based on aggregated data, also considering that we used structurally aggregation invariant MPT models for which parameter estimates tend to be fairly robust (Erdfelder et al., 2024).

### Results

**Fig. 3:**
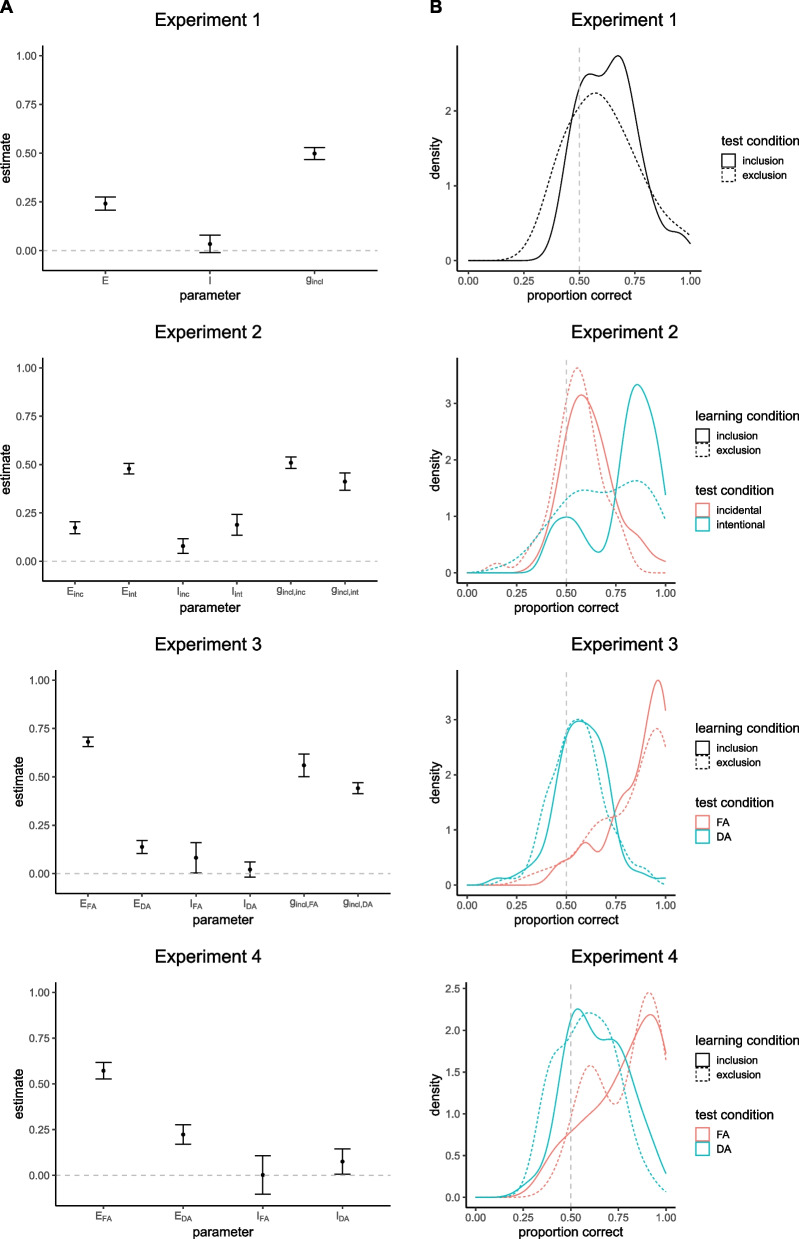
Parameter estimates of the baseline model (**A**) and kernel density plots of the individual proportions of correct responses (action repetitions in the inclusion condition and action alternations in the exclusion condition) for the different experimental conditions (**B**). *Notes.* Error bars represent 95% CIs. incl = inclusion, excl = exclusion, inc = incidental, int = intentional, FA = full attention, DA = divided attention

Participants on average correctly *reproduced* the action with which they previously produced the effect image reencountered at test with a proportion of .63 (*SD* = .13) in the inclusion condition and correctly *alternated* their response with a proportion of .61 (*SD* = .16) in the exclusion condition. Aggregated response frequencies are shown in Table 2 in Appendix A. Parameter estimates of the baseline MPT model are displayed in Fig. 3A. Specific values are shown in Table 3 in Appendix B. Additionally, kernel density plots of the individual proportions of correct responses (an action repetition at test in the inclusion condition and an action alternation at test in the exclusion condition) are displayed in Fig. 3B.^3^ The baseline model showed good model fit according to a log-likelihood ratio test (see Table 1). Exploratory conditional likelihood-ratio tests (see Table 1) revealed that fixing parameter $E$ to zero significantly worsened model fit, suggesting the involvement of explicit memory. Restricting parameter $I$ to zero did not significantly worsen model fit, thus not yielding evidence for the involvement of implicit memory. In line with our expectation, applying an equality constraint on parameters E and I significantly worsened model fit, suggesting a larger contribution of explicit than implicit memory (cf. Fig. 3A).

**Table 1:**
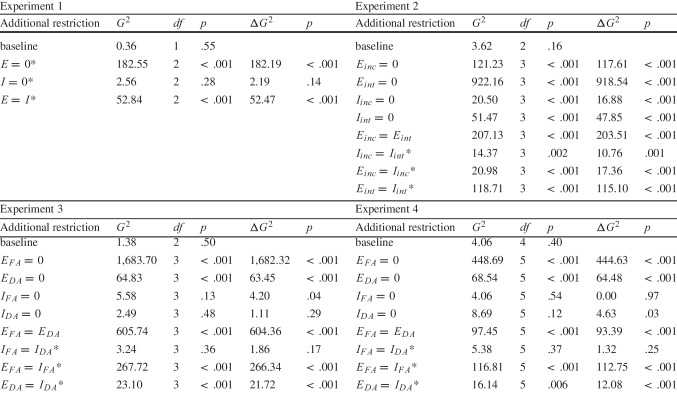
Model fit indices ($G^2$) for the baseline model and nested models with additional parameter restrictions and differences in model fit indices ($\Delta G^2$) of the nested models compared to the baseline model (nested model - baseline model) for all experiments

*Notes.* *Exploratory test. incl = inclusion, excl = exclusion, inc = incidental, int = intentional, FA = full attention, DA = divided attention. $\Delta df = 1$ for all model comparisons

## Experiment 2: Incidental vs. intentional learning

In Experiment 2, participants either incidentally or intentionally learned action–effect relations.

### Methods

There were two learning conditions (incidental and intentional). The incidental learning condition was identical to Experiment 1. In the intentional learning condition, participants were instructed to remember the effect images and corresponding actions, and were informed about the later memory test. There were again two test conditions (inclusion and exclusion).

#### Participants

A new set of participants was recruited via the Prolific panel (https://www.prolific.com) and received a compensation of £2.10 for the approximately 14-min experiment. They were again prescreened to speak English fluently and to have normal or corrected-to-normal vision and no color vision deficiency. A simulation-based a priori power analysis conducted in multiTree v0.47 (Moshagen, 2010) for detecting a difference of .05 between the explicit memory parameters of the incidental ($E_{inc}$) and intentional ($E_{int}$) learning condition with 80% power given a significance level of 5% yielded a desired sample size of 61 participants per experimental condition. To account for possible data exclusions, we collected data from 65 participants per condition. Given four conditions, this resulted in a target sample size of 260 participants. However, the data of *N* = 262 participants was collected before data collection was stopped. In total, 69 participants were excluded due to error rates in the learning phase larger than 20% (2) and responding with the same key in the test phase in more than 95% of trials (2). We further excluded participants in the incidental learning condition who reported being aware of the later memory test at the end of the experiment (38) and participants in the intentional learning condition who reported being unaware of the later memory test (27).^4^ This resulted in a final sample size of *N* = 193 participants (45 participants in the inclusion incidental condition, 52 in the exclusion incidental condition, 43 in the inclusion intentional condition, and 53 in the exclusion intentional condition). Participants were on average 32.10 years old (*SD* = 10.54, range = 18–77), 99 participants were women and 94 were men, 172 participants were right-handed, 14 were left-handed, and seven were ambidextrous.

#### Design

The experiment employed a two-factorial (learning condition: incidental vs. intentional $\times$ test condition: inclusion vs. exclusion) between-participants design. The incidental learning condition was identical to Experiment 1, that is, participants received no instructions to remember the effect images and corresponding actions and were not informed that their memory would later be tested. In the intentional learning condition, participants were instructed to try to remember the effect images and corresponding actions, as their memory would later be tested, before the learning phase. Test conditions were identical to Experiment 1. Participants were randomly assigned to the experimental conditions.

#### Materials and procedure

Materials were identical to Experiment 1. The procedure was also identical to Experiment 1, except that participants in the intentional learning condition received corresponding instructions before the learning phase and we asked participants whether they were aware of the later memory test during the learning phase at the end of the experiment.

#### Data analysis

We analyzed the data the same way as in Experiment 1, using the same basic MPT model. However, given the more complex experimental design, we extended the model to consist of eight trees (four per learning condition) and 12 parameters (six per learning condition). Parameter $E_{A, inc}$ denotes the probability to retrieve action A from explicit memory in the incidental learning condition, parameter $E_{L, inc}$ denotes the probability to retrieve action L from explicit memory in the incidental learning condition, parameter $E_{A, int}$ denotes the probability to retrieve action A from explicit memory in the intentional learning condition, parameter $E_{L, int}$ denotes the probability to retrieve action L from explicit memory in the intentional learning condition, parameter $I_{A, inc}$ denotes the probability to retrieve action A from implicit memory in the incidental learning condition, parameter $I_{L, inc}$ denotes the probability to retrieve action L from implicit memory in the incidental learning condition, parameter $I_{A, int}$ denotes the probability to retrieve action A from implicit memory in the intentional learning condition, and parameter $I_{L, int}$ denotes the probability to retrieve action L from implicit memory in the intentional learning condition. Parameters $g_{incl, inc}$, $g_{incl, int}$, $g_{excl, inc}$, and $g_{excl, int}$ are guessing parameters denoting the probability to respond with action A in the different experimental conditions, respectively, if retrieval from explicit or implicit memory is unsuccessful.

To make the model identifiable, we again imposed theoretically derived restrictions on the model parameters. First, we applied equality constraints on the explicit and implicit memory parameters within the learning conditions, thus assuming action invariance (i.e., no difference in explicit and implicit memory between actions A and L) and estimating only two parameters for explicit ($E_{inc}$ and $E_{int}$) and implicit ($I_{inc}$ and $I_{int}$) memory retrieval. Second, we fixed $g_{excl, inc}$ and $g_{excl, int}$ to the stochastic guessing probability of .5 given two possible responses in the test phase, thus assuming unbiased guessing in the exclusion conditions. Note that this slightly deviates from the preregistration, where we noted to also fix $g_{incl, inc}$ and $g_{incl, int}$ to .5. However, this yielded an inadequate model fit due to some biased guessing in the inclusion conditions. An additional post hoc simulated identifiability analysis suggested that the model remains identifiable without this restriction, and relaxing it yielded a good model fit. We thus report the results of the better-fitting model, but note that this did not affect the qualitative interpretation of the results.

In line with the testing approach in Experiment 1, we tested whether the contribution of explicit memory differed between the incidental and intentional learning condition by fitting a nested model with an equality constraint applied to parameters $E_{inc}$ and $E_{int}$ and conducting a model comparison. We further tested whether there was general evidence for the contribution of explicit and/or implicit memory in the respective learning condition in a similar manner, by fitting nested models, with the explicit and implicit memory parameters in the respective condition fixed to zero. As additional exploratory analyses, we further tested for a difference in the contribution of implicit memory between learning conditions ($I_{inc}$ = $I_{int}$), and for the comparison of explicit and implicit memory contributions within learning conditions ($E_{inc}$ = $I_{inc}$ and $E_{int}$ = $I_{int}$).

### Results

Participants on average correctly *reproduced* actions corresponding to the presented effect images at test with a proportion of .62 (*SD* = .13) in the inclusion incidental learning condition and .79 (*SD* = .17) in the inclusion intentional learning condition, and correctly *alternated* their response with a proportion of .55 (*SD* = .12) in the exclusion incidental learning condition and .69 (*SD* = .20) in the exclusion intentional learning condition. The baseline model showed good model fit according to a log-likelihood ratio test (see Table 1). Conditional likelihood-ratio tests (see Table 1) revealed that fixing either parameters $E_{inc}$ and $E_{int}$ to zero significantly worsened model fit, suggesting the involvement of explicit memory in both learning conditions. In line with our expectation, applying an equality constraint on these parameters also significantly worsened model fit, suggesting the contribution of explicit memory to be larger in the intentional than in the incidental learning condition (cf. Fig. 3A). Regarding implicit memory, fixing either parameters $I_{inc}$ and $I_{int}$ to zero significantly worsened model fit, suggesting the involvement of implicit memory in both the incidental and intentional learning conditions. In addition, an exploratory test revealed that applying an equality constraint on these parameters significantly worsened model fit, suggesting the contribution of implicit memory to be larger in the intentional learning condition (cf. Fig. 3A). However, further exploratory tests showed that applying an equality constraint on the explicit and implicit memory parameters within learning conditions ($E_{inc}$ = $I_{inc}$ or $E_{int}$ = $I_{int}$) significantly worsened model fit, suggesting the contribution of explicit memory to be larger than that of implicit memory in both conditions (cf. Fig. 3A).

## Experiment 3: Intentional learning at full vs. divided attention

In Experiment 3, participants intentionally learned action–effect relations under full or divided attention.

### Methods

There were two learning conditions (full attention and divided attention). The full attention condition was identical to the intentional learning condition of Experiment 2. Thus, in the learning phase, participants only had to perform the single task of producing effects through correct actions. In the divided attention condition, participants had to perform an additional listening task in which they had to monitor a list of auditorily presented digit recordings and react to target sequences of three odd digits in a row (Craik, 1982; Craik et al., 2018; Jacoby, 1991). There were again two test conditions (inclusion and exclusion).

#### Participants

A new set of participants was recruited via the Prolific panel (https://www.prolific.com) and received a compensation of £2.55 for the approximately 17-min experiment. They were prescreened to speak English fluently, to have normal or corrected-to-normal vision and no color vision deficiency, and to have no hearing difficulties. A simulation-based a priori power analysis conducted in multiTree v0.47 (Moshagen, 2010) for detecting a difference of .05 between the explicit memory parameters of the full attention ($E_{FA}$) and divided attention ($E_{DA}$) learning condition with 80% power given a significance level of 5% yielded a desired sample size of 122 participants per test condition. To account for possible data exclusion, we oversampled by 20%, thus collecting data from 148 participants per test condition (*N* = 296). In total, 131 participants were excluded due to experiencing technical problems or distractions (9), error rates in the generation task in the learning phase larger than 30% (1), a low proportion of hits (less than 50%) in the listening task in the learning phase (102), and reporting being unaware of the later memory test at the end of the experiment (27).^5^ This resulted in a final sample size of *N* = 165 participants (80 participants in the inclusion condition and 85 in the exclusion condition). Participants were on average 32.90 years old (*SD* = 11.70, range = 19–70), 71 participants were women and 94 were men, 146 participants were right-handed, 16 were left-handed, and three were ambidextrous.

#### Design

The experiment employed a two-factorial (learning condition: full vs. divided attention $\times$ test condition: inclusion vs. exclusion) mixed design. Test condition was a between-participants factor and conditions were identical to Experiments 1 and 2. Learning condition was a within-participants factor. The full attention condition was identical to the intentional learning condition in Experiment 2, that is, participants only performed one task (the generation task, as in Experiments 1 and 2). In the divided attention condition, participants simultaneously conducted the generation task and a listening task. The full and divided attention conditions were implemented as blocks of 20 action–effect episodes each, with the order of blocks being implemented as an additional between-participants condition for counterbalancing. Participants were randomly assigned to the between-participants conditions.

#### Materials and procedure

Object images were identical to Experiments 1 and 2. The learning phase was largely identical to that of Experiment 2, except for the additional listening task (Craik, 1982; Craik et al., 2018; Jacoby, 1991) in the divided attention condition. In the listening task, participants had to monitor a list of random digits presented auditorily at a rate of 1.5 s via headphones and detect target sequences of three odd numbers in a row by pressing the spacebar. The randomization was constrained such that there was a minimum of one and a maximum of five even numbers between two target sequences. Digit recordings were created using the Narakeet AI voice generator (https://www.narakeet.com/create/ai-voice-generator.html, Video Puppet Limited, 2024) and were spoken by a female voice with an American accent. Each recording had a duration of 1 s. If participants responded between the onset of the last digit of a target sequence and before the onset of the next digit, their response was counted as a hit; otherwise, it was counted as a miss. Participants were instructed that it is important that they do not miss a target sequence and that they should try to be as accurate as possible in both the generation and listening tasks but avoid the generation task from disrupting their performance in the listening task. At the beginning of the experiment, participants could listen to a test recording (the same voice as for the digits saying “test”) and were instructed to adjust their volume so they could hear it loudly but comfortably. The learning phase was split into two blocks, with each block being assigned to a learning condition. Each learning block consisted of 60 trials (with 20 effect images, each produced three times through the same action). The required action was counterbalanced within blocks. Each block was preceded by four practice trials (not repeated) to familiarize participants with the procedure. Participants were instructed beforehand that they should try to remember the object images they produce and the action (i.e., the keypress) with which they produced them, and that their memory would later be tested (as in the intentional learning condition of Experiment 2). The test phase was identical to the one of Experiments 1 and 2.

#### Data analysis

We analyzed the data using the same MPT model as in Experiment 2, except that parameters referring to the incidental and intentional learning conditions now referred to the full (FA) and divided attention (DA) conditions. We also applied the same parameter restrictions as in Experiment 2. Again, note that this slightly deviates from the preregistration, where we noted to also fix $g_{incl, FA}$ and $g_{incl, DA}$ to .5. However, this again yielded an inadequate model fit due to some biased guessing in the inclusion conditions. We thus report the results of the better-fitting model, but this again did not affect the qualitative interpretation of the results.

In line with the testing approach in Experiments 1 and 2, we tested whether the contribution of explicit memory differed between the full and divided attention condition by fitting a nested model with an equality constraint applied to parameters $E_{FA}$ and $E_{DA}$ and conducting a model comparison. To test whether divided attention during encoding reduced the involvement of explicit memory to a greater extent than that of implicit memory, we compared a model with an equality constraint on parameters $E_{FA}$ and $E_{DA}$ with a model with an equality constraint on parameters $I_{FA}$ and $I_{FA}$. Because these models are not nested, we compared their model fit via the difference in the Akaike Information criterion (AIC) (Akaike, 1973), which, given that both models contain the same number of parameters, is directly proportional to the models’ likelihood. We considered one of the models to yield a substantially better fit than the other, given a difference in AIC ($\Delta$AIC) of at least 10 (cf. Burnham & Anderson, 2002). We further tested whether there was general evidence for the contribution of explicit and/or implicit memory in the respective learning condition in a similar manner, by fitting nested models, with the explicit and implicit memory parameters in the respective condition fixed to zero. Additionally, we tested for a difference in the contribution of implicit memory between learning conditions ($I_{FA}$ = $I_{DA}$), and, as exploratory analyses, for a difference in explicit and implicit memory contributions within learning conditions ($E_{FA}$ = $I_{FA}$ and $E_{DA}$ = $I_{DA}$).

### Results

Participants on average correctly *reproduced* actions corresponding to the presented effect images at test with a proportion of .85 (*SD* = .15) in the inclusion full attention condition and .58 (*SD* = .14) in the inclusion divided attention condition, and correctly *alternated* their response with a proportion of .83 (*SD* = .18) in the exclusion full attention condition and .56 (*SD* = .13) in the exclusion divided attention condition. The baseline model showed good model fit according to a log-likelihood ratio test (see Table 1). Conditional likelihood-ratio tests (see Table 1) revealed that fixing either parameters $E_{FA}$ and $E_{DA}$ to zero significantly worsened model fit, suggesting the involvement of explicit memory in both the full and divided attention conditions. In line with our expectation, applying an equality constraint on these parameters also significantly worsened model fit, suggesting the contribution of explicit memory to be reduced in the divided compared to the full attention condition (cf. Fig. 3A). Regarding implicit memory, fixing parameter $I_{FA}$ to zero significantly worsened model fit, suggesting the involvement of implicit memory in the full attention condition. However, a likelihood-ratio test showed that the nested model with this restriction still exhibited good model fit. Thus, evidence for the contribution of implicit memory in the full attention condition was somewhat ambiguous. Fixing parameter $I_{DA}$ to zero did not significantly worsen model fit, thus yielding no evidence for the involvement of implicit memory in the divided attention condition. An exploratory test revealed that applying an equality constraint on parameters $I_{FA}$ and $I_{DA}$ did not significantly worsen model fit, thus yielding no evidence for the contribution of implicit memory to differ between the full and divided attention conditions. Other exploratory tests further showed that applying an equality constraint on the explicit and implicit memory parameters within learning conditions ($E_{FA}$ = $I_{DA}$ or $E_{FA}$ = $I_{DA}$) significantly worsened model fit, suggesting the contribution of explicit memory to be larger than that of implicit memory in both conditions (cf. Fig. 3A). We further tested whether divided attention at encoding reduces the contribution of explicit memory to a greater extent than that of implicit memory. To do this, we conducted a model comparison between a nested model with an equality constraint on the explicit memory parameters ($E_{FA}$ = $E_{DA}$) and a nested model with an equality constraint on the implicit memory parameters ($I_{FA}$ = $I_{DA}$). This analysis revealed that the model with an equality constraint on the implicit memory parameters (AIC = 13.24) fit substantially better than the model with an equality constraint on the explicit memory parameters (AIC = 615.74, $\Delta AIC$ = 602.51). In line with our expectation, this suggests that the contribution of explicit memory varied more strongly between the full and divided attention condition than the contribution of implicit memory.

Many participants performed poorly in the listening task, which necessitated the exclusion of a rather large number of participants according to the preregistered protocol (102 participants with a proportion of hits below .5). The non-excluded participants had an average proportion of hits of .82 (*SD* = .15). In Experiment 4, we simplified the listening task to evaluate the robustness of the results.

## Experiment 4: Intentional learning at full vs. divided attention with a simplified divided attention task

In Experiment 4, we simplified the listening task in the divided attention condition. Otherwise, the experiment was largely identical to Experiment 3, and results largely converged.

### Methods

In the listening task, participants had to monitor a list of high- and low-pitched tones and react to three high-pitched tones in a row. Thus, there were again two learning (full attention and divided attention) and two test (inclusion and exclusion) conditions.

#### Participants

A new set of participants was recruited via the Prolific panel (https://www.prolific.com) and received a compensation of £2.55 for the approximately 17-min experiment. They were again prescreened to speak English fluently, to have normal or corrected-to-normal vision and no color vision deficiency, and to have no hearing difficulties. A simulation-based a priori power analysis conducted in multiTree v0.47 (Moshagen, 2010) for detecting a difference of .10 between the explicit memory parameters of the full attention ($E_{FA}$) and divided attention ($E_{DA}$) learning condition with 80% power given a significance level of 5% yielded a desired sample size of 32 participants per test condition. To account for possible data exclusion, we oversampled by 30%, thus collecting data from 42 participants per test condition. In line with the preregistered protocol, we collected data from an additional 21 participants (*N* = 105) to reach the target sample size after data exclusions. In total, 41 participants were excluded due to experiencing technical problems (3), not understanding instructions (1), a low proportion of hits (less than 50%) in the listening task in the learning phase (24), and due to reporting being unaware of the later memory test at the end of the experiment (13).^6^ This resulted in a final sample size of *N* = 64 participants (33 participants in the inclusion condition and 31 in the exclusion condition). Participants were on average 33.38 years old (*SD* = 11.74, range = 18–63), 28 participants were women, 35 were men, and one was non-binary, 54 participants were right-handed, eight were left-handed, and two were ambidextrous.

#### Design

The experiment’s design was identical to the one of Experiment 3 and was thus again a two-factorial (learning condition: full vs. divided attention $\times$ test condition: inclusion vs. exclusion) mixed design. Test condition was a between-participants factor and learning condition was a within-participants factor. Again, the full and divided attention conditions were implemented as blocks of 20 action-effect episodes each, with the order of blocks being implemented as an additional between-participants condition for counterbalancing. Participants were randomly assigned to the between-participants conditions.

#### Materials and procedure

Object images were identical to Experiments 1-3. The experimental procedure was identical to the one of Experiment 3, except that we used a simplified listening task in the divided attention condition. Instead of monitoring a list of odd and even digits, in the listening task of Experiment 4, participants had to monitor a list of low- and high-pitched tones presented at a rate of 1.5 s and detect target sequences of three high-pitched tones in a row by pressing the spacebar. Similar to Experiment 3, the randomization was constrained such that there was a minimum of one and a maximum of five low-pitched tones between two target sequences. Tones were sine tones with a frequency of 300 Hz (low-pitched) and 800 Hz (high-pitched) with a duration of 0.5 s, a level of -3 dBFS, and a sample rate of 44.1 kHz, created with the Audio Check Single Sine Tone Generator (https://www.audiocheck.net/audiofrequencysignalgenerator_sinetone.php, Pigeon, 2022). At the beginning of the experiment, participants could listen to both tones and were instructed to adjust their volume so they could hear them loudly but comfortably.

#### Data analysis

Data analysis was identical to Experiment 3. However, in Experiment 4, the model with all guessing parameters (i.e., $g_{incl, FA}$, $g_{incl, DA}$, $g_{excl, FA}$, and $g_{excl, DA}$) fixed to .5, as preregistered, yielded good model fit, so we report the results of this model.

### Results

Participants on average correctly *reproduced* actions corresponding to the presented effect images at test with a proportion of .79 (*SD* = .19) in the inclusion full attention condition and .64 (*SD* = .16) in the inclusion divided attention condition, and correctly *alternated* their response with a proportion of .79 (*SD* = .17) in the exclusion full attention condition and .58 (*SD* = .15) in the exclusion divided attention condition. The baseline model showed good model fit according to a log-likelihood ratio test (see Table 1). Conditional likelihood-ratio tests (see Table 1) revealed that fixing either parameters $E_{FA}$ and $E_{DA}$ to zero significantly worsened model fit, suggesting the involvement of explicit memory in both the full and divided attention conditions. In line with our expectation, applying an equality constraint on these parameters also significantly worsened model fit, suggesting the contribution of explicit memory to be reduced in the divided compared to the full attention condition (cf. Fig. 3A). Regarding implicit memory, fixing parameter $I_{FA}$ to zero did not significantly worsen model fit, thus yielding no evidence for the involvement of implicit memory in the full attention condition. Fixing parameter $I_{DA}$ to zero significantly worsened model fit, suggesting the involvement of implicit memory in the divided attention condition. However, a likelihood-ratio test showed that the nested model with this restriction still exhibited good model fit. Thus, evidence for the contribution of implicit memory in the divided attention condition was somewhat ambiguous. An exploratory test revealed that applying an equality constraint on parameters $I_{FA}$ and $I_{DA}$ did not significantly worsen model fit, thus yielding no evidence for the contribution of implicit memory to differ between the full and divided attention conditions. Other exploratory tests further showed that applying an equality constraint on the explicit and implicit memory parameters within learning conditions ($E_{FA}$ = $I_{DA}$ or $E_{FA}$ = $I_{DA}$) significantly worsened model fit, suggesting the contribution of explicit to be larger than that of implicit memory in both conditions (cf. Fig. 3A). We further tested whether divided attention at encoding reduces the contribution of explicit memory to a greater extent than that of implicit memory. Therefore, we again conducted a model comparison between a nested model with an equality constraint on the explicit memory parameters ($E_{FA}$ = $E_{DA}$) and a nested model with an equality constraint on the implicit memory parameters ($I_{FA}$ = $I_{DA}$). This analysis revealed that the model with an equality constraint on the implicit memory parameters (AIC = 11.38) fit substantially better than the model with an equality constraint on the explicit memory parameters (AIC = 103.45, $\Delta$AIC = 92.07). In line with our expectation, this suggests that the contribution of explicit memory varied more strongly between the full and divided attention condition than the contribution of implicit memory. Regarding the listening task, the non-excluded participants had an average proportion of hits of .86 (*SD* = .12).

## Discussion

In four experiments, we investigated the contribution of explicit and implicit memory processes to the learning of action–effect relations, focusing on early learning with few repetitions of action–effect episodes. We further provide a new and now validated methodological approach to directly distinguish between explicit and implicit memory representations of action–effect relations. Our findings suggest that, at least in early learning stages studied here, action–effect relations are primarily represented in explicit memory. In fact, unambiguous evidence for the involvement of implicit memory processes was only found in one of the experiments (Experiment 2), and also there the contribution of implicit memory was much smaller than that of explicit memory. We further found memory to be facilitated by intentional (compared to incidental) learning and to be reduced given divided (compared to full) attention during encoding, with these factors primarily affecting explicit memory.

These findings challenge ideomotor-theoretical reasoning, which largely assumes action–effect relations to be stored in implicit memory, likely in procedural memory (Squire, 2004), thus evoking automatic action tendencies not affording conscious reasoning (Greenwald, 1970; Hommel et al., 2001; James, 1890; Kunde, 2004). They are, however, in line with recent evidence pointing towards an involvement of explicit memory processes (Custers, 2023; Janczyk et al., 2024; Schreiner et al., 2025a; Sun et al., 2020; see also Schreiner & Kunde, 2025b; Schreiner & Kunde, 2025).

Our findings provide the first direct evidence for the primary involvement of explicit memory processes in the learning of action–effect relations. We deem it likely that the early bottom-up learning (through experiencing action–effect episodes) studied here rests on episodic memory representations enabling rapid learning (Horner & Burgess, 2013; Ngo et al., 2019; Schreiner et al., 2023). From these representations, propositional knowledge may be extracted over time and with repeated encounters via interactions between the episodic and semantic memory systems, as suggested by complementary learning systems theory (Kumaran et al., 2016; McClelland et al., 1995; O’Reilly et al., 2014).

One may argue that the memory test we employed is a direct test of memory, as we explicitly asked participants to reproduce or alternate the action associated with a given effect, and may therefore naturally probe explicit memory. Note, however, that task distinctions should not be equated with process distinctions (Jacoby, 1991) and that such direct retrieval instructions were actually identified as critical for the process dissociation procedure (Yonelinas & Jacoby, 2012).

While we found the learning of action–effect relations to primarily rely on explicit memory, explicit and implicit memory representations are not necessarily mutually exclusive. In fact, many dual process models assume that they can be stored and operate in parallel (Gawronski & Bodenhausen, 2006; Jacoby, 1991; Yonelinas, 2002). Indeed, in Experiment 2 we found evidence for the involvement of both explicit and implicit memory processes, with both processes being facilitated by intentional learning (although explicit memory to a considerably stronger extent). However, in Experiments 3 and 4, which also involved intentional learning, the involvement of implicit memory processes could not be clearly replicated. This warrants further scrutiny in future research. One may argue that, given that our test was not speeded, fast automatic action tendencies elicited by implicit memory representations could be overruled by conscious reasoning based on explicit memory representations (cf. Toth, 1996; Yonelinas & Jacoby, 1994). In this case, however, a weakening of explicit memory traces should increase the manifestation of implicit ones. This was not observed in Experiments 3 and 4. While divided attention during encoding substantially reduced the contribution of explicit memory compared to full attention, it did not significantly increase the involvement of implicit memory.

Note that we focused here on early learning stages in which each action–effect episode was only encountered three times. It is conceivable that the involvement of implicit memory increases with repeated encounters of action–effect episodes. The learning of action–effect relations may first entail the accumulation of explicit memory representations, which eventually feed into implicit memory to enable automated behavior (see also Ericsson, 1998, 2008; Logan, 1988). Therefore, initially explicit memory representations may (partly) be transferred to the implicit memory system through system-level consolidation processes (Dudai et al., 2015), possibly along the hippocampal-striatal axis (Pennartz et al., 2011). While previous studies did not directly dissociate explicit and implicit memory representations, many studies which were interpreted as yielding evidence in favor of implicit representations of action–effect relations involved a small number of action–effect relations that had to be learned and a high number of repetitions (such as 100) of action–effect episodes (e.g., Elsner and Hommel, 2001; Herwig and Waszak, 2009; Hommel et al., 2003). These studies may therefore have provided beneficial conditions for the expression of implicit memory processes (see also Gawronski and Bodenhausen, 2006), whereas others may have provided beneficial conditions for explicit memory process, such as low cognitive load (e.g., Sun et al., 2020) or a high number of action–effect relations (Schreiner et al., 2025a; Schreiner & Kunde, 2025). Our results, however, suggest that explicit memory representations of action–effect relations may be a prerequisite for the (later) formation of implicit representations. This may be the basic mechanism underlying the acquisition of skills. Such a mechanism would suggest a shift from explicit to implicit memory representations with increasing experience, which should be tested in future research.

Finally, one may ask to what extent the rather simple keypress actions we employed in our experiments relate to actual causal interactions with our environment. First, in the highly mechanized and digitalized world we live in, we can often produce complex outcomes through simple actions. For example, when operating a computer, we can open an image through a simple key press or mouse click. Similarly, when operating a 3D printer, we can initiate the production of a physical object through a simple command, such as pressing a key. Second, even simple actions like pressing a key (e.g., on a piano to produce a tone) are already quite complex from a motor control perspective (Turvey & Fonseca, 2009) and do not fundamentally differ from more complex actions (Bozzacchi et al., 2012) such as gestures or grasping actions. We therefore believe that our results also extend to more complex actions, although there, of course, additional processes may be at play. For example, for actions that consist of several discrete movements, such as playing a melody on a piano, these different steps (and their respective effects) may have to be integrated into a higher-level action representation (cf. Moeller and Frings, 2019). However, for this to be achieved, first, the individual action–effect relations have to be learned. Third, other studies investigating the learning of action–effect relations also employed simple keypress actions (e.g., Elsner and Hommel, 2001; Janczyk et al., 2024; Kunde, 2004; Sun et al., 2020) and the present set of experiments is therefore highly comparable to these previous studies. Finally, our experiments focus on the early learning of action–effect relations, where little to no prior information about these relations is available. This relates to, for example, learning in new and unfamiliar environments, the acquisition of new skills that are very different from already learned ones, and the acquisition of action–effect relations in infants, which possess little or no prior knowledge about action–effect relations but are already able to acquire action–effect knowledge (Verschoor et al., 2013).

## Figures and Tables

**Table 3:**
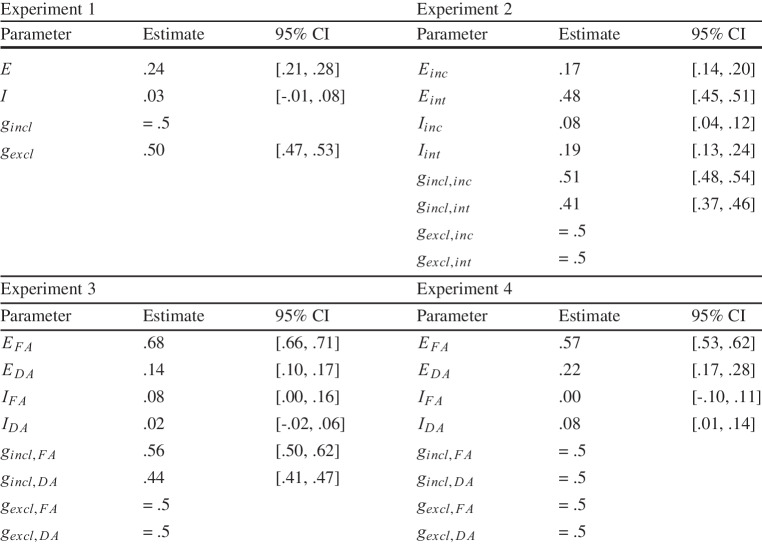
Parameter estimates of the baseline model in all experiments

*Notes*. Subscripts indicate experimental conditions: inc = incidental, int = intentional, FA = full attention, DA = divided attention (learning conditions), incl = inclusion, excl = exclusion (test conditions). E = probability to retrieve action from explicit memory (estimated jointly for actions A and L, i.e., an equality constraint was applied to parameters $E_{A}$ and $E_{L}$), $I =$ probability to retrieve action from implicit memory (estimated jointly for actions A and L, i.e., an equality constraint was applied to parameters $I_{A}$ and $I_{L}$), $g_{incl} =$ probability to guess and respond with action A in the inclusion condition, $g_{excl}=$ probability to guess and respond with action A in the exclusion condition. An equal sign in the “Estimate” column indicates that the parameter was fixed to the indicated value

## Data Availability

The data and study materials for all experiments are available at the Open Science Framework (https://doi.org/10.17605/OSF.IO/WS9E4).

## Appendix A: Response frequencies

**Table 2:** Aggregated response frequencies in all experiments

Experiment	Action	Learning condition	Test condition	Response	*n*	Frequency
1	A	incidental	inclusion	A	43	544
1	A	incidental	inclusion	L	43	316
1	L	incidental	inclusion	A	43	314
1	L	incidental	inclusion	L	43	546
1	A	incidental	exclusion	A	35	269
1	A	incidental	exclusion	L	35	431
1	L	incidental	exclusion	A	35	420
1	L	incidental	exclusion	L	35	280
2	A	incidental	inclusion	A	45	564
2	A	incidental	inclusion	L	45	336
2	L	incidental	inclusion	A	45	349
2	L	incidental	inclusion	L	45	551
2	A	incidental	exclusion	A	52	476
2	A	incidental	exclusion	L	52	564
2	L	incidental	exclusion	A	52	589
2	L	incidental	exclusion	L	52	451
2	A	intentional	inclusion	A	43	646
2	A	intentional	inclusion	L	43	214
2	L	intentional	inclusion	A	43	150
2	L	intentional	inclusion	L	43	710
2	A	intentional	exclusion	A	53	312
2	A	intentional	exclusion	L	53	748
2	L	intentional	exclusion	A	53	715
2	L	intentional	exclusion	L	53	345
3	A	full attention	inclusion	A	80	697
3	A	full attention	inclusion	L	80	103
3	L	full attention	inclusion	A	80	131
3	L	full attention	inclusion	L	80	669
3	A	full attention	exclusion	A	85	147
3	A	full attention	exclusion	L	85	703
3	L	full attention	exclusion	A	85	704
3	L	full attention	exclusion	L	85	146
3	A	divided attention	inclusion	A	80	423
3	A	divided attention	inclusion	L	80	377
3	L	divided attention	inclusion	A	80	298
3	L	divided attention	inclusion	L	80	502
3	A	divided attention	exclusion	A	85	386
3	A	divided attention	exclusion	L	85	464
3	L	divided attention	exclusion	A	85	488
3	L	divided attention	exclusion	L	85	362
4	A	full attention	inclusion	A	33	259
4	A	full attention	inclusion	L	33	71
4	L	full attention	inclusion	A	33	70
4	L	full attention	inclusion	L	33	260
4	A	full attention	exclusion	A	31	64
4	A	full attention	exclusion	L	31	246
4	L	full attention	exclusion	A	31	241
4	L	full attention	exclusion	L	31	69
4	A	divided attention	inclusion	A	33	200
4	A	divided attention	inclusion	L	33	130
4	L	divided attention	inclusion	A	33	107
4	L	divided attention	inclusion	L	33	223
4	A	divided attention	exclusion	A	31	133
4	A	divided attention	exclusion	L	31	177
4	L	divided attention	exclusion	A	31	184
4	L	divided attention	exclusion	L	31	126

*Notes*. *n* = number of participants in each experimental condition. Experiments 1 and 2: Each action 20 times per experimental condition, Experiments 3 and 4: Each action ten times per experimental condition

## Footnotes

1 Note that an alternative model structure, where implicit memory processes come before explicit memory processes and can be overridden by the latter, is mathematically equivalent to the shown model.

2 Note that results remained virtually unchanged when instead fixing $g_{incl}$ to .5.

3 These tables and figures show the respective results for all experiments.

4 Results were robust regardless of the awareness exclusion criteria.

5 Results were robust regardless of the awareness exclusion criterion. They were further robust when using a less strict exclusion criterion based on performance in the listening task (excluding participants with a proportion of hits less than 20%), thus only excluding 58 participants on this basis, except that in this case, restricting $I_{DA}$ to zero also led to significantly worse model fit.

6 Again, results were robust regardless of the awareness exclusion criterion. They were also again robust when using a less strict exclusion criterion based on performance in the listening task (excluding participants with a proportion of hits less than 20%), thus only excluding 16 participants on this basis, except that in this case, restricting $I_{DA}$ to zero did not lead to significantly worse model fit.

## Code availability

Analysis scripts and experiment code are available at the Open Science Framework (https://doi.org/10.17605/OSF.IO/WS9E4).

## Declarations

### Open Practices Statement

The data and materials for all experiments are available at https://doi.org/10.17605/OSF.IO/WS9E4. Experiment 1 was not preregistered and the conducted tests were thus exploratory. Experiment 2 was preregistered at https://doi.org/10.17605/OSF.IO/Z9BYM. Experiment 3 was preregistered at https://doi.org/10.17605/OSF.IO/JHWUN. Experiment 4 was preregistered at https://doi.org/10.17605/OSF.IO/TRHUZ. For Experiments 22 and 3, there was a minor deviation from the preregistration (freely estimating the $g_{incl}$ parameters to achieve good model fit), which is clearly identified. Post hoc analyses are clearly identified as such.
