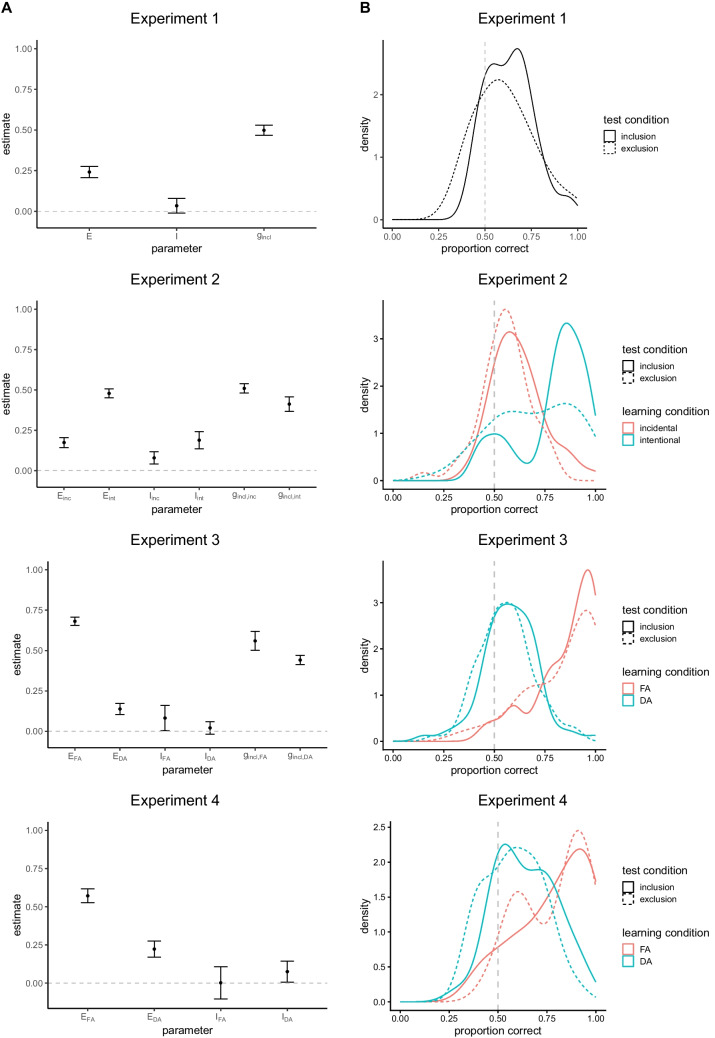# Correction: The representational nature of action–effect relations: A memory process dissociation approach

**DOI:** 10.3758/s13423-026-02870-2

**Published:** 2026-02-17

**Authors:** Marcel R. Schreiner, Wilfried Kunde

**Affiliations:** Department of Psychology III, Julius-Maximilians-Universität Würzburg, Röntgenring 11, 97070 Würzburg, Germany

**Correction: Psychonomic Bulletin & Review (2026) 33:46**

10.3758/s13423-025-02794-3

This paper published with an incorrect figure (with interchanged legend titles). The correct figure is presented below.